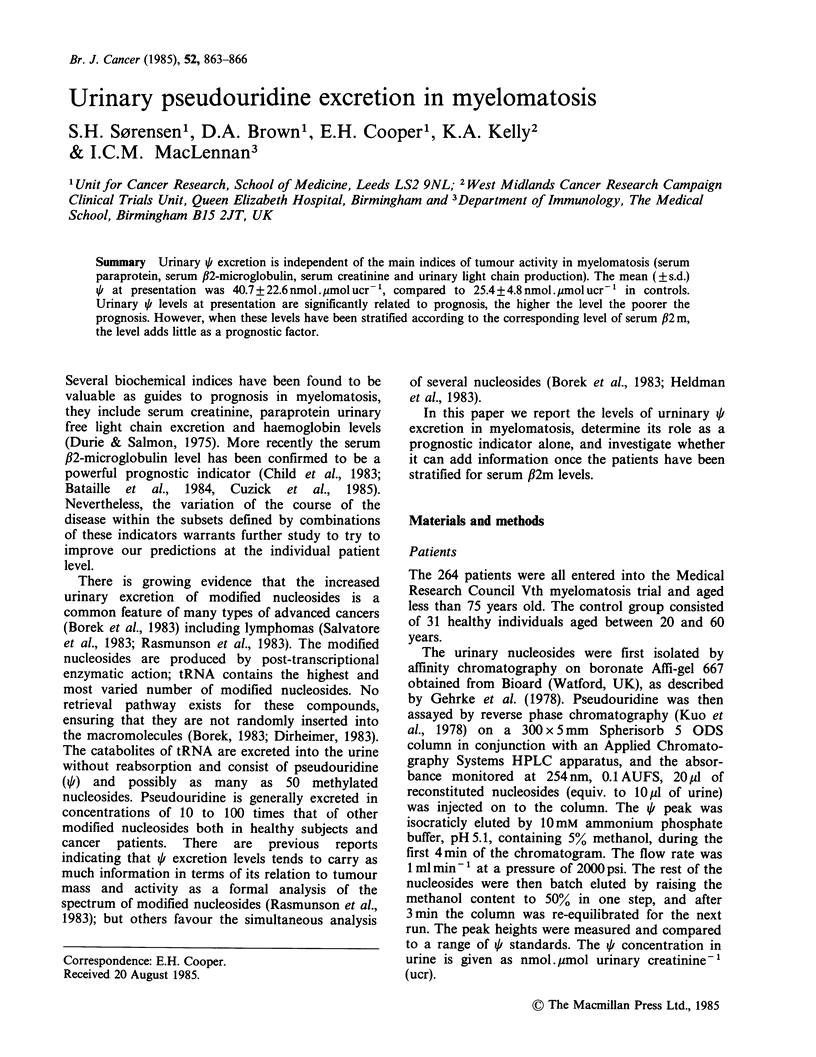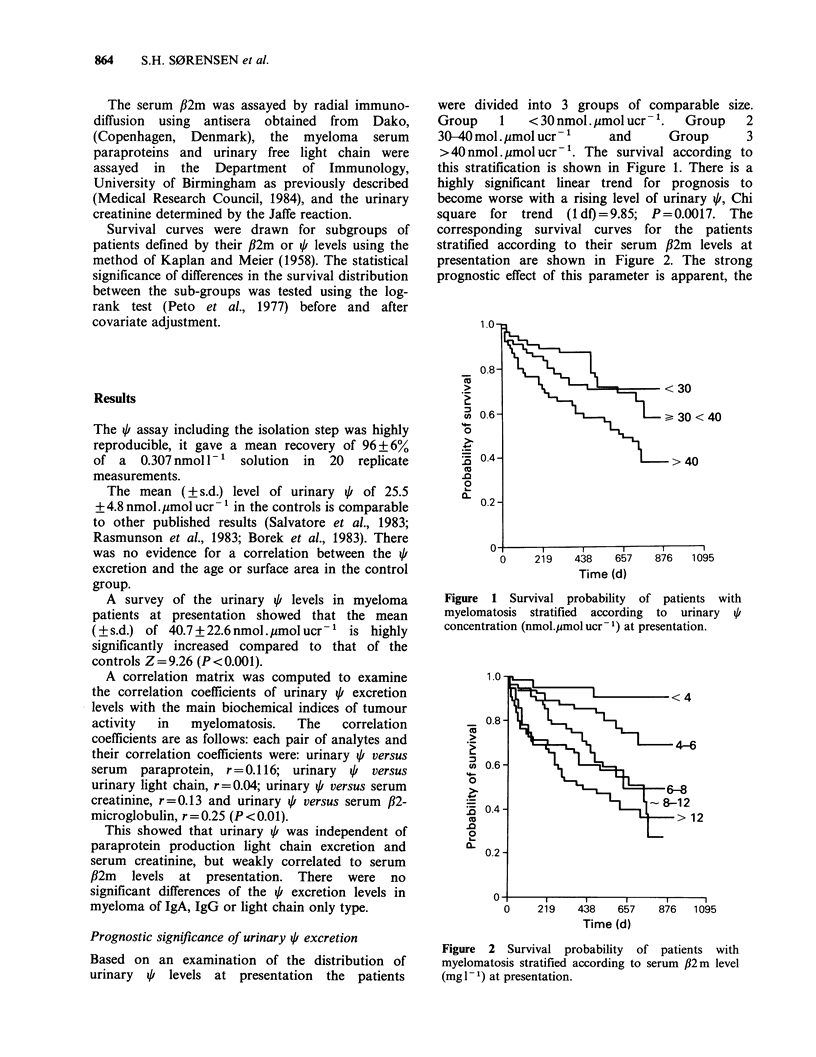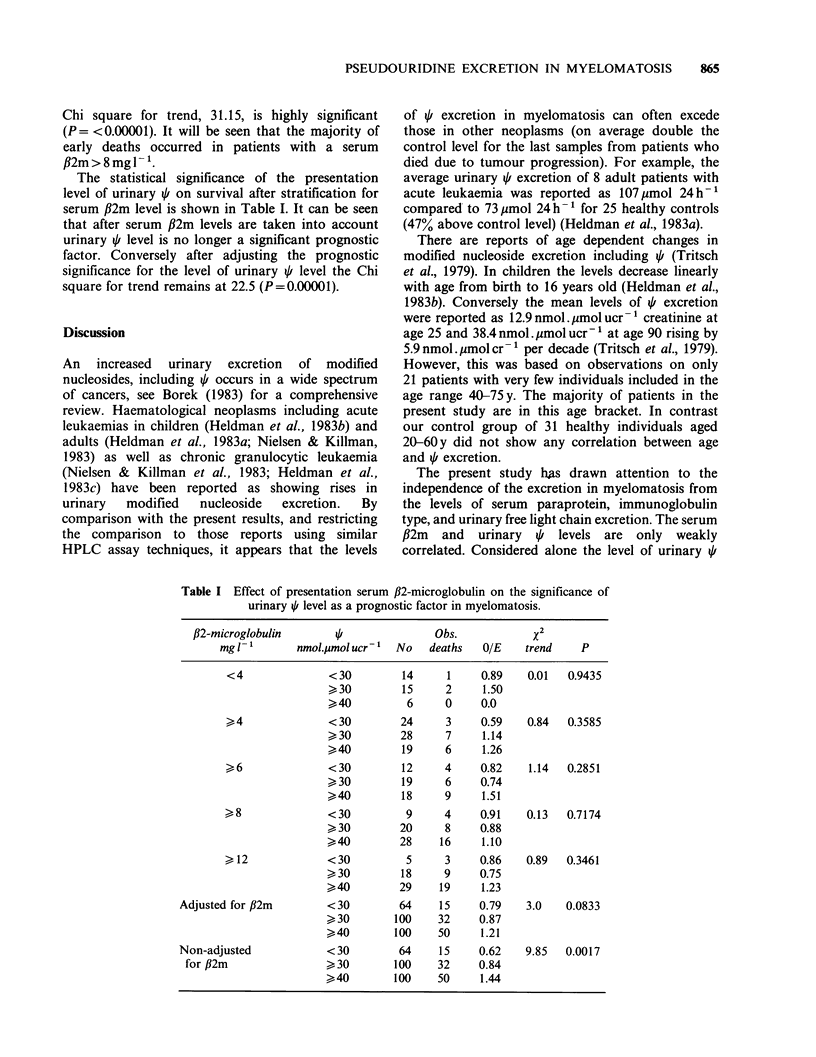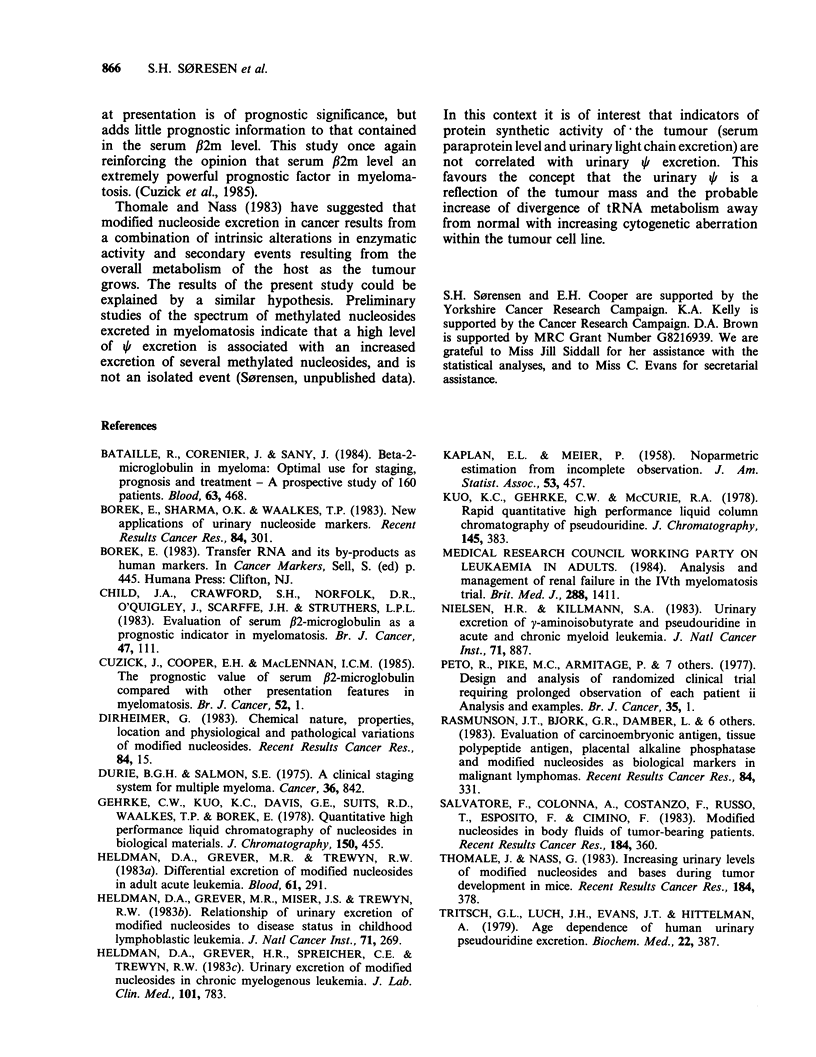# Urinary pseudouridine excretion in myelomatosis.

**DOI:** 10.1038/bjc.1985.270

**Published:** 1985-12

**Authors:** S. H. Sørensen, D. A. Brown, E. H. Cooper, K. A. Kelly, I. C. MacLennan

## Abstract

Urinary psi excretion is independent of the main indices of tumour activity in myelomatosis (serum paraprotein, serum beta 2-microglobulin, serum creatinine and urinary light chain production). The mean (+/- s.d.) psi at presentation was 40.7 +/- 22.6 nmol. mumol ucr-1, compared to 25.4 +/- 4.8 nmol. mumol ucr-1 in controls. Urinary psi levels at presentation are significantly related to prognosis, the higher the level the poorer the prognosis. However, when these levels have been stratified according to the corresponding level of serum beta 2m, the level adds little as a prognostic factor.


*Br. J. Cancer* (1985), **52**, 863–866

### Urinary pseudouridine excretion in myelomatosis

S.H. Sørensen[1], D.A. Brown[1], E.H. Cooper[1], K.A. Kelly[2] & I.C.M. MacLennan[3]

[1]*Unit for Cancer Research, School of Medicine, Leeds LS2 9NL;* [2]*West Midlands Cancer Research Campaign Clinical Trials Unit, Queen Elizabeth Hospital, Birmingham and* [3]*Department of Immunology, The Medical School, Birmingham B15 2JT, UK*

**Summary** Urinary $\psi$ excretion is independent of the main indices of tumour activity in myelomatosis (serum paraprotein, serum $\beta$2-microglobulin, serum creatinine and urinary light chain production). The mean ($\pm$s.d.) $\psi$ at presentation was $40.7 \pm 22.6$ nmol.$\mu$mol ucr$^{-1}$, compared to $25.4 \pm 4.8$ nmol.$\mu$mol ucr$^{-1}$ in controls. Urinary $\psi$ levels at presentation are significantly related to prognosis, the higher the level the poorer the prognosis. However, when these levels have been stratified according to the corresponding level of serum $\beta$2 m, the level adds little as a prognostic factor.

Several biochemical indices have been found to be valuable as guides to prognosis in myelomatosis, they include serum creatinine, paraprotein urinary free light chain excretion and haemoglobin levels (Durie & Salmon, 1975). More recently the serum $\beta$2-microglobulin level has been confirmed to be a powerful prognostic indicator (Child *et al.*, 1983; Bataille *et al.*, 1984, Cuzick *et al.*, 1985). Nevertheless, the variation of the course of the disease within the subsets defined by combinations of these indicators warrants further study to try to improve our predictions at the individual patient level.

There is growing evidence that the increased urinary excretion of modified nucleosides is a common feature of many types of advanced cancers (Borek *et al.*, 1983) including lymphomas (Salvatore *et al.*, 1983; Rasmunson *et al.*, 1983). The modified nucleosides are produced by post-transcriptional enzymatic action; tRNA contains the highest and most varied number of modified nucleosides. No retrieval pathway exists for these compounds, ensuring that they are not randomly inserted into the macromolecules (Borek, 1983; Dirheimer, 1983). The catabolites of tRNA are excreted into the urine without reabsorption and consist of pseudouridine ($\psi$) and possibly as many as 50 methylated nucleosides. Pseudouridine is generally excreted in concentrations of 10 to 100 times that of other modified nucleosides both in healthy subjects and cancer patients. There are previous reports indicating that $\psi$ excretion levels tends to carry as much information in terms of its relation to tumour mass and activity as a formal analysis of the spectrum of modified nucleosides (Rasmunson *et al.*, 1983); but others favour the simultaneous analysis of several nucleosides (Borek *et al.*, 1983; Heldman *et al.*, 1983).

In this paper we report the levels of urninary $\psi$ excretion in myelomatosis, determine its role as a prognostic indicator alone, and investigate whether it can add information once the patients have been stratified for serum $\beta$2m levels.

### Materials and methods

### *Patients*

The 264 patients were all entered into the Medical Research Council Vth myelomatosis trial and aged less than 75 years old. The control group consisted of 31 healthy individuals aged between 20 and 60 years.

The urinary nucleosides were first isolated by affinity chromatography on boronate Affi-gel 667 obtained from Bioard (Watford, UK), as described by Gehrke *et al.* (1978). Pseudouridine was then assayed by reverse phase chromatography (Kuo *et al.*, 1978) on a 300×5 mm Spherisorb 5 ODS column in conjunction with an Applied Chromatography Systems HPLC apparatus, and the absorbance monitored at 254 nm, 0.1 AUFS, 20 $\mu$l of reconstituted nucleosides (equiv. to 10 $\mu$l of urine) was injected on to the column. The $\psi$ peak was isocratically eluted by 10 mM ammonium phosphate buffer, pH 5.1, containing 5% methanol, during the first 4 min of the chromatogram. The flow rate was 1 ml min$^{-1}$ at a pressure of 2000 psi. The rest of the nucleosides were then batch eluted by raising the methanol content to 50% in one step, and after 3 min the column was re-equilibrated for the next run. The peak heights were measured and compared to a range of $\psi$ standards. The $\psi$ concentration in urine is given as nmol.$\mu$mol urinary creatinine$^{-1}$ (ucr).

Correspondence: E.H. Cooper.
Received 20 August 1985.

© The Macmillan Press Ltd., 1985

The serum $\beta$2m was assayed by radial immunodiffusion using antisera obtained from Dako, (Copenhagen, Denmark), the myeloma serum paraproteins and urinary free light chain were assayed in the Department of Immunology, University of Birmingham as previously described (Medical Research Council, 1984), and the urinary creatinine determined by the Jaffe reaction.

Survival curves were drawn for subgroups of patients defined by their $\beta$2m or $\psi$ levels using the method of Kaplan and Meier (1958). The statistical significance of differences in the survival distribution between the sub-groups was tested using the log-rank test (Peto *et al.*, 1977) before and after covariate adjustment.

### Results

The $\psi$ assay including the isolation step was highly reproducible, it gave a mean recovery of $96 \pm 6\%$ of a 0.307 nmol l$^{-1}$ solution in 20 replicate measurements.

The mean ($\pm$s.d.) level of urinary $\psi$ of 25.5 $\pm$4.8 nmol.$\mu$mol ucr$^{-1}$ in the controls is comparable to other published results (Salvatore *et al.*, 1983; Rasmunson *et al.*, 1983; Borek *et al.*, 1983). There was no evidence for a correlation between the $\psi$ excretion and the age or surface area in the control group.

A survey of the urinary $\psi$ levels in myeloma patients at presentation showed that the mean ($\pm$s.d.) of $40.7 \pm 22.6$ nmol.$\mu$mol ucr$^{-1}$ is highly significantly increased compared to that of the controls $Z = 9.26$ ($P < 0.001$).

A correlation matrix was computed to examine the correlation coefficients of urinary $\psi$ excretion levels with the main biochemical indices of tumour activity in myelomatosis. The correlation coefficients are as follows: each pair of analytes and their correlation coefficients were: urinary $\psi$ *versus* serum paraprotein, $r = 0.116$; urinary $\psi$ *versus* urinary light chain, $r = 0.04$; urinary $\psi$ *versus* serum creatinine, $r = 0.13$ and urinary $\psi$ *versus* serum $\beta$2-microglobulin, $r = 0.25$ ($P < 0.01$).

This showed that urinary $\psi$ was independent of paraprotein production light chain excretion and serum creatinine, but weakly correlated to serum $\beta$2m levels at presentation. There were no significant differences of the $\psi$ excretion levels in myeloma of IgA, IgG or light chain only type.

### *Prognostic significance of urinary $\psi$ excretion*

Based on an examination of the distribution of urinary $\psi$ levels at presentation the patients were divided into 3 groups of comparable size. Group 1 $<30$ nmol.$\mu$mol ucr$^{-1}$. Group 2 30–40 mol.$\mu$mol ucr$^{-1}$ and Group 3 $>40$ nmol.$\mu$mol ucr$^{-1}$. The survival according to this stratification is shown in Figure 1. There is a highly significant linear trend for prognosis to become worse with a rising level of urinary $\psi$, Chi square for trend (1 df) = 9.85; $P = 0.0017$. The corresponding survival curves for the patients stratified according to their serum $\beta$2m levels at presentation are shown in Figure 2. The strong prognostic effect of this parameter is apparent, the

**Figure 1** Survival probability of patients with myelomatosis stratified according to urinary $\psi$ concentration (nmol.$\mu$mol ucr$^{-1}$) at presentation.

**Figure 2** Survival probability of patients with myelomatosis stratified according to serum $\beta$2m level (mg l$^{-1}$) at presentation.

Chi square for trend, 31.15, is highly significant ($P = <0.00001$). It will be seen that the majority of early deaths occurred in patients with a serum $\beta$2m$>8\,mg\,l^{-1}$.

The statistical significance of the presentation level of urinary $\psi$ on survival after stratification for serum $\beta$2m level is shown in Table I. It can be seen that after serum $\beta$2m levels are taken into account urinary $\psi$ level is no longer a significant prognostic factor. Conversely after adjusting the prognostic significance for the level of urinary $\psi$ level the Chi square for trend remains at 22.5 ($P = 0.00001$).

### Discussion

An increased urinary excretion of modified nucleosides, including $\psi$ occurs in a wide spectrum of cancers, see Borek (1983) for a comprehensive review. Haematological neoplasms including acute leukaemias in children (Heldman *et al.*, 1983*b*) and adults (Heldman *et al.*, 1983*a*; Nielsen & Killman, 1983) as well as chronic granulocytic leukaemia (Nielsen & Killman *et al.*, 1983; Heldman *et al.*, 1983*c*) have been reported as showing rises in urinary modified nucleoside excretion. By comparison with the present results, and restricting the comparison to those reports using similar HPLC assay techniques, it appears that the levels of $\psi$ excretion in myelomatosis can often excede those in other neoplasms (on average double the control level for the last samples from patients who died due to tumour progression). For example, the average urinary $\psi$ excretion of 8 adult patients with acute leukaemia was reported as $107\,\mu mol\ 24\,h^{-1}$ compared to $73\,\mu mol\ 24\,h^{-1}$ for 25 healthy controls (47% above control level) (Heldman *et al.*, 1983*a*).

There are reports of age dependent changes in modified nucleoside excretion including $\psi$ (Tritsch *et al.*, 1979). In children the levels decrease linearly with age from birth to 16 years old (Heldman *et al.*, 1983*b*). Conversely the mean levels of $\psi$ excretion were reported as $12.9\,nmol.\mu mol\,ucr^{-1}$ creatinine at age 25 and $38.4\,nmol.\mu mol\,ucr^{-1}$ at age 90 rising by $5.9\,nmol.\mu mol\,cr^{-1}$ per decade (Tritsch *et al.*, 1979). However, this was based on observations on only 21 patients with very few individuals included in the age range 40–75 y. The majority of patients in the present study are in this age bracket. In contrast our control group of 31 healthy individuals aged 20–60 y did not show any correlation between age and $\psi$ excretion.

The present study has drawn attention to the independence of the excretion in myelomatosis from the levels of serum paraprotein, immunoglobulin type, and urinary free light chain excretion. The serum $\beta$2m and urinary $\psi$ levels are only weakly correlated. Considered alone the level of urinary $\psi$

**Table I** Effect of presentation serum $\beta$2-microglobulin on the significance of urinary $\psi$ level as a prognostic factor in myelomatosis.

| *β2-microglobulin mg l⁻¹* | *ψ nmol.μmol ucr⁻¹* | *No* | *Obs. deaths* | *0/E* | *χ² trend* | *P* |
|---|---|---|---|---|---|---|
| <4 | <30 | 14 | 1 | 0.89 | 0.01 | 0.9435 |
| | ≥30 | 15 | 2 | 1.50 | | |
| | ≥40 | 6 | 0 | 0.0 | | |
| ≥4 | <30 | 24 | 3 | 0.59 | 0.84 | 0.3585 |
| | ≥30 | 28 | 7 | 1.14 | | |
| | ≥40 | 19 | 6 | 1.26 | | |
| ≥6 | <30 | 12 | 4 | 0.82 | 1.14 | 0.2851 |
| | ≥30 | 19 | 6 | 0.74 | | |
| | ≥40 | 18 | 9 | 1.51 | | |
| ≥8 | <30 | 9 | 4 | 0.91 | 0.13 | 0.7174 |
| | ≥30 | 20 | 8 | 0.88 | | |
| | ≥40 | 28 | 16 | 1.10 | | |
| ≥12 | <30 | 5 | 3 | 0.86 | 0.89 | 0.3461 |
| | ≥30 | 18 | 9 | 0.75 | | |
| | ≥40 | 29 | 19 | 1.23 | | |
| Adjusted for β2m | <30 | 64 | 15 | 0.79 | 3.0 | 0.0833 |
| | ≥30 | 100 | 32 | 0.87 | | |
| | ≥40 | 100 | 50 | 1.21 | | |
| Non-adjusted for β2m | <30 | 64 | 15 | 0.62 | 9.85 | 0.0017 |
| | ≥30 | 100 | 32 | 0.84 | | |
| | ≥40 | 100 | 50 | 1.44 | | |

at presentation is of prognostic significance, but adds little prognostic information to that contained in the serum $\beta$2m level. This study once again reinforcing the opinion that serum $\beta$2m level an extremely powerful prognostic factor in myelomatosis. (Cuzick *et al.*, 1985).

Thomale and Nass (1983) have suggested that modified nucleoside excretion in cancer results from a combination of intrinsic alterations in enzymatic activity and secondary events resulting from the overall metabolism of the host as the tumour grows. The results of the present study could be explained by a similar hypothesis. Preliminary studies of the spectrum of methylated nucleosides excreted in myelomatosis indicate that a high level of $\psi$ excretion is associated with an increased excretion of several methylated nucleosides, and is not an isolated event (Sørensen, unpublished data). In this context it is of interest that indicators of protein synthetic activity of the tumour (serum paraprotein level and urinary light chain excretion) are not correlated with urinary $\psi$ excretion. This favours the concept that the urinary $\psi$ is a reflection of the tumour mass and the probable increase of divergence of tRNA metabolism away from normal with increasing cytogenetic aberration within the tumour cell line.

S.H. Sørensen and E.H. Cooper are supported by the Yorkshire Cancer Research Campaign. K.A. Kelly is supported by the Cancer Research Campaign. D.A. Brown is supported by MRC Grant Number G8216939. We are grateful to Miss Jill Siddall for her assistance with the statistical analyses, and to Miss C. Evans for secretarial assistance.

### References

BATAILLE, R., CORENIER, J. & SANY, J. (1984). Beta-2-microglobulin in myeloma: Optimal use for staging, prognosis and treatment – A prospective study of 160 patients. *Blood*, **63**, 468.

BOREK, E., SHARMA, O.K. & WAALKES, T.P. (1983). New applications of urinary nucleoside markers. *Recent Results Cancer Res.*, **84**, 301.

BOREK, E. (1983). Transfer RNA and its by-products as human markers. In *Cancer Markers*, Sell, S. (ed) p. 445. Humana Press: Clifton, NJ.

CHILD, J.A., CRAWFORD, S.H., NORFOLK, D.R., O'QUIGLEY, J., SCARFFE, J.H. & STRUTHERS, L.P.L. (1983). Evaluation of serum $\beta$2-microglobulin as a prognostic indicator in myelomatosis. *Br. J. Cancer*, **47**, 111.

CUZICK, J., COOPER, E.H. & MACLENNAN, I.C.M. (1985). The prognostic value of serum $\beta$2-microglobulin compared with other presentation features in myelomatosis. *Br. J. Cancer*, **52**, 1.

DIRHEIMER, G. (1983). Chemical nature, properties, location and physiological and pathological variations of modified nucleosides. *Recent Results Cancer Res.*, **84**, 15.

DURIE, B.G.H. & SALMON, S.E. (1975). A clinical staging system for multiple myeloma. *Cancer*, **36**, 842.

GEHRKE, C.W., KUO, K.C., DAVIS, G.E., SUITS, R.D., WAALKES, T.P. & BOREK, E. (1978). Quantitative high performance liquid chromatography of nucleosides in biological materials. *J. Chromatography*, **150**, 455.

HELDMAN, D.A., GREVER, M.R. & TREWYN, R.W. (1983*a*). Differential excretion of modified nucleosides in adult acute leukemia. *Blood*, **61**, 291.

HELDMAN, D.A., GREVER, M.R., MISER, J.S. & TREWYN, R.W. (1983*b*). Relationship of urinary excretion of modified nucleosides to disease status in childhood lymphoblastic leukemia. *J. Natl Cancer Inst.*, **71**, 269.

HELDMAN, D.A., GREVER, H.R., SPREICHER, C.E. & TREWYN, R.W. (1983*c*). Urinary excretion of modified nucleosides in chronic myelogenous leukemia. *J. Lab. Clin. Med.*, **101**, 783.

KAPLAN, E.L. & MEIER, P. (1958). Noparmetric estimation from incomplete observation. *J. Am. Statist. Assoc.*, **53**, 457.

KUO, K.C., GEHRKE, C.W. & MCCURIE, R.A. (1978). Rapid quantitative high performance liquid column chromatography of pseudouridine. *J. Chromatography*, **145**, 383.

MEDICAL RESEARCH COUNCIL WORKING PARTY ON LEUKAEMIA IN ADULTS. (1984). Analysis and management of renal failure in the IVth myelomatosis trial. *Brit. Med. J.*, **288**, 1411.

NIELSEN, H.R. & KILLMANN, S.A. (1983). Urinary excretion of $\gamma$-aminoisobutyrate and pseudouridine in acute and chronic myeloid leukemia. *J. Natl Cancer Inst.*, **71**, 887.

PETO, R., PIKE, M.C., ARMITAGE, P. & 7 others. (1977). Design and analysis of randomized clinical trial requiring prolonged observation of each patient ii Analysis and examples. *Br. J. Cancer*, **35**, 1.

RASMUNSON, J.T., BJORK, G.R., DAMBER, L. & 6 others. (1983). Evaluation of carcinoembryonic antigen, tissue polypeptide antigen, placental alkaline phosphatase and modified nucleosides as biological markers in malignant lymphomas. *Recent Results Cancer Res.*, **84**, 331.

SALVATORE, F., COLONNA, A., COSTANZO, F., RUSSO, T., ESPOSITO, F. & CIMINO, F. (1983). Modified nucleosides in body fluids of tumor-bearing patients. *Recent Results Cancer Res.*, **184**, 360.

THOMALE, J. & NASS, G. (1983). Increasing urinary levels of modified nucleosides and bases during tumor development in mice. *Recent Results Cancer Res.*, **184**, 378.

TRITSCH, G.L., LUCH, J.H., EVANS, J.T. & HITTELMAN, A. (1979). Age dependence of human urinary pseudouridine excretion. *Biochem. Med.*, **22**, 387.